# circABCB10 Promotes Malignant Progression of Gastric Cancer Cells by Preventing the Degradation of MYC

**DOI:** 10.1155/2021/4625033

**Published:** 2021-12-14

**Authors:** Yingchun Zhang, Yong Zhou, Fang Wei

**Affiliations:** Department of General Surgery, The Fourth Affiliated Hospital of China Medical University, Shenyang, Liaoning 110032, China

## Abstract

**Objective:**

To investigate the role of circABCB10 in gastric cancer and the molecular mechanism of promoting malignant progression of gastric cancer cells by preventing the degradation of MYC by hsa-miR-1252-5p.

**Methods:**

The expression of circABCB10 in gastric cancer tissues and cells was detected by real-time quantitative PCR. MTT, Transwell, clone formation, and TUNEL assay were used to detect the effects of circABCB10 on the proliferation, invasion, and apoptosis of gastric cancer cells. A subcutaneous tumor-bearing model was established to study the inhibitory effect of knockdown circABCB10 on gastric cancer proliferation. The dual luciferase reporter gene assay and RNA pull-down assay were used to verify the regulatory effect of circABCB10 on miR-1252-5p and the regulatory effect of miR-1252-5p on MYC.

**Results:**

Compared with paracancerous tissues and gastric mucosal epithelial cells, the expression of circABCB10 was significantly increased in human gastric cancer tissues and gastric cancer cells. circABCB10 knockout significantly decreased cell viability and invasion ability and promoted cell apoptosis (*P* < 0.01). Subcutaneous tumor-bearing experiments in nude mice demonstrated that circABCB10 knockdown inhibited the proliferation of gastric cancer cells. circABCB10 can act as a sponge for miR-1252-5p in gastric cancer cells. Meanwhile, MYC is the target gene of miR-1252-5p. Overexpression of miR-1252-5p and knockdown of MYC reversed the promoting effect of circABCB10 on gastric cancer.

**Conclusion:**

circABCB10 can promote the proliferation, invasion, and clonal formation of gastric cancer cells by targeting miR-1252-5p and upregulating the expression of MYC. circABCB10/miR-1252-5p/MYC constitutes the regulatory mechanism of ceRNA.

## 1. Introduction

Gastric cancer is one of the most common malignant tumors of the digestive system [1]. The incidence and mortality of gastric cancer were the fifth and the third in malignant tumor incidence and mortality, respectively. At present, surgery, radiotherapy, chemotherapy, and immunotherapy are the main treatment methods for gastric cancer [2]. However, the exact pathogenesis and invasion and metastasis mechanism of gastric cancer are still not very clear, leading to the failure of the ideal early diagnosis and treatment of gastric cancer. Therefore, there is an urgent need to find new biomarkers and precise therapeutic targets related to the occurrence and prognosis of gastric cancer [3, 4].

Circular RNAs (circRNAs) are a kind of covalent closed-loop RNA formed by special cleavage [5]. circRNAs lack the 3′ and 5′ ends of conventional linear RNAs and are named for their circular structure. circRNAs have been found to have multiple biological functions [6]. circRNAs can act as molecular “sponges” to adsorb microRNA (miRNA), participate in transcription and posttranscriptional regulation, and serve as templates to guide translation, etc. Studies have shown that a large number of circRNAs are involved in the regulation of tumor genesis and development [7]. circRNAs are expected to be new tumor markers and therapeutic targets. In addition, because circRNA has the “sponge” characteristics of miRNA molecules, it can be used as the carrier of exogenous miRNA, so it also has a good prospect of clinical application [8]. The role of circRNA in gastric cancer has also attracted more and more attention. In some studies, 180 differentially expressed circRNAs were identified by sequencing the cancer tissues and matched normal gastric mucosa epithelial tissues of 3 patients with gastric cancer. It has previously been reported that circRNA circABCB10 promotes breast cancer proliferation and progression by targeting miR-1271 [9]. circABCB10 also promotes proliferation and migration of non-small-cell lung cancer cells by regulating the miR-1252/FoxR2 axis [10]. circABCB10 is involved in paclitaxel resistance in breast cancer via the let-7a-5p/DUSP7 axis [11]. However, the role of circABCB10 in the progression of gastric cancer remains unclear. Therefore, this study aims to detect the expression of circABCB10 in gastric cancer tissues and cells, as well as its effect on the biological behavior of gastric cancer cells, and further investigate the relevant molecular mechanisms.

c-MYC is a transcription regulator encoded by the protooncogene MYC and plays an important role in promoting tumorigenesis, maintaining tumor cell growth, proliferation and differentiation, angiogenesis and apoptosis [12]. Abnormal expression of c-MYC has been found in most malignant tumors, including breast cancer, colon cancer, cervical cancer, myeloid leukemia, melanoma, osteosarcoma, glioblastoma, small-cell lung cancer, and medulloblastoma [13]. Through the gradual understanding of the carcinogenic properties of abnormal c-MYC expression, c-MYC has become a therapeutic target for many malignant tumors. However, the role of MYC in gastric cancer still needs to be further studied, especially its upstream regulatory mechanism.

The purpose of this study was to analyze the expression of circABCB10 in gastric cancer tissues and cells. At the same time, the effects of circABCB10 on the proliferation, invasion, and cell migration of gastric cancer cells were analyzed. The molecular mechanism of circABCB10 regulating the biological function of gastric cancer by competitively binding with miR-1252-5p to affect the expression of MYC has been studied. This study provides a new idea for understanding the pathogenesis of gastric cancer.

## 2. Methods

### 2.1. The Tissue and Clinical Information of Gastric Cancer Was Collected

A total of 20 patients with gastric cancer who received surgical treatment in our hospital from September 2018 to May 2020 were selected as the research subjects. All the patients did not receive radiotherapy or chemotherapy before surgery, and all the data were pathologically confirmed to be gastric cancer. Gastric cancer and its adjacent tissues (more than 5 cm away from the cancer foci without cancer cells) were taken for experimental study. All patients signed an informed consent form. The study plan was approved by the Medical Ethics Committee of the Fourth Affiliated Hospital of China Medical University.

### 2.2. Cell Culture

Gastric cancer cell lines and gastric mucosal epithelial cells GES-1 were cultured in RPMI-1640 (containing 10% fetal bovine serum, 100 U/mL penicillin, and 100 *μ*g/mL streptomycin). All the cells were cultured in an incubator at 37°C with 5% CO_2_ and were subcultured after the cells grew to 70%–80%. The cells used in the experiment were all logarithmic growth cells.

### 2.3. Cell Transfection

Cells were inoculated into culture plates overnight to achieve a density of about 30% to 40%. 5 *μ*L Lipofectamine™ 2000 was added to 250 *μ*L Opti-MEM medium. Another 1.5 mL RNA-free EP tube containing 250 *μ*L Opti-MEM medium was added to 5 *μ*L siRNA or miRNA. Mix gently and let sit for 5 min. Mix the solution from the two tubes and let stand for 20 min. Absorb and discard the original medium in the culture plate and replace it with serum-free medium. Add the transfection mixture to the appropriate hole. After 4–6 h, the medium with serum was replaced, and consumables without RNA enzymes were used in the transfection process.

### 2.4. Real-Time Fluorescent Quantitative PCR (qRT-PCR) Was Used to Detect Gene Expression Levels in Gastric Tissues and Cells

Total RNA was extracted from gastric cancer tissues, paracancerous tissues, gastric cancer cells, and gastric epithelial cells by TRIzol method. Follow the instructions of the TaqMan RNA reverse transcription kit. 4 ng total RNA was reverse-transcribed into cDNA. In CFX96 real-time quantitative PCR instrument, U6 or GAPDH was used as internal parameters to quantify the expression level of target. QRT-PCR was performed using SYBR Green PCR premix reagent. Internal reference GAPDH upstream primer: 5′-CCCACTTCTCTCTAAGGAGAAT-3′, downstream primer: 5′-TACACGAAAGCAATGCTATCAC-3′. 2^−ΔΔCt^ method was used to reflect the relative gene expression level.

### 2.5. CCK-8 Assay Was Used to Detect Cell Viability

MKN-45 and MKN28 cells were collected. 5000 cells/well were inoculated in 96-well plates. Each group is provided with 3 duplicate holes. It was cultured in a cell incubator. CCK-8 detection was performed at 72 h. Add 10 *μ*L CCK-8 reagent to each well. Incubate in cell incubator for 2 h. The optical density value at 450 nm was detected with a microplate analyzer [D(450 nm)].

### 2.6. EdU Experiment

Cells were processed and counted, and 8000–15000 cells were evenly spread into 96-well plates in each well, and three duplicate wells were set. The reagent A was diluted in a 1000 : 1 ratio in a serum-containing medium. 100 *µ*l per well was added to the corresponding well of the 96-well plate and incubated in a 37°C cell incubators for 2 h. Each group was added with the cell fixation solution prepared with 4% paraformaldehyde in PBS and incubated for 30 min at room temperature in a shaking table. The stationary solution was discarded and 50 *µ*l 2 mg/mL glycine solution was added to each well. Incubate them in a shaker for 5 min and discard them. Add 100 ul penetrant (0.5% TritonX-100) to each well. 1 × Apollo solution, 100 *µ*l per well, was incubated with shaker at room temperature for 30 min. After 30 min, the reaction solution was discarded and 100 *µ*l of penetrant was added to each well. Wash it on the shaker for 3 times, 10 min each. Then, add 100 ul of methanol solution to each well. After abandoning PBS, 1 × DAPI reaction solution was added to each well. The reaction solution was discarded after 30 minutes of dark incubation in a shaker. The 96-well plate was placed under an inverted fluorescence microscope and photographed for image acquisition.

### 2.7. Transwell Experiment

Dilute the gel according to the matrix: serum-free medium = 1 : 5. 40 *µ*l of each compartment is evenly added to the upper layer. Place the chamber in a cell incubator for 4–6 h to solidify it. The transfected cells were digested with trypsin, and serum-containing culture was added to terminate digestion. After gently beating and evenly mixing, the cell suspension was transferred to a 5 ml centrifuge tube at 800 rpm for 5 min. The supernatant was discarded, and PBS was added to resuspend the cells and centrifuged again. PBS was discarded; the cells were resuspended and counted by adding serum-free medium into the centrifuge tube. The cell suspension was prepared with serum-free medium at 3 × 10^5^/mL. Add 600 *µ*l medium containing 20% fetal bovine serum to the lower compartment. Add 200 *µ*l of the prepared cell suspension to the upper layer. The chambers were transferred to cell incubators for 24–48 h. Remove the compartment and gently wipe off the upper cells with a cotton swab. PBS was washed twice, and 600 *µ*l methanol was taken to fix the cells in the lower layer of the chamber for 30 min. The chamber was dried and stained with crystal violet for 30 min. Photographs were taken for counting under a microscope.

### 2.8. Clone Formation Experiment

MKN-45 and MKN28 cells were seeded into 6-well plates (500 cells/well). Each group was set with three duplicate wells and cultured in 37°C incubator for 10 ∼ 12 days. The liquid is changed every 2∼3 days. The culture of clone cells was stopped when they grew to a suitable size. PBS was used for cleaning 3 times. The cells were fixed at 4°C with methanol for 15 min. PBS was used for cleaning 3 times. Add 1 mL crystal violet to each well and stain for 20 min. Rinse with water until no background exists. Count the number of cell clones, and the rate of clone formation (%) = (number of clone/total number of inoculated cells) × 100%.

### 2.9. The Apoptosis of Gastric Cancer Cells in Each Group Was Detected by TUNEL Assay

After 0.1%Trition X-100 penetration, the cells were added with protease K and allowed to stand for 20 min to inactivate DNA and RNA. Rinse again and add TUNEL reaction solution. Incubate in 37°C incubator for 1 h in darkness. After washing with PBS for 3 times, DAPI was added and rinsed with PBS again for 3 times. Mounting medium is used to seal tablets. Take pictures under a microscope. Apoptosis rate (%) = number of positive cells in each field/total number of all cells in the field × 100%.

### 2.10. Xenograft Tumor Model

In this study, gastric cancer cells were transfected with lentivirus that knocked down circaBCB10. Lentivirus transfection experiments with circABCB10 interference were divided into two groups: control group: LV-si-NC and knockdown group: LV-si-circABCB10. Human gastric cancer cells are infected by a lentivirus carrying the circABCB10 interfering gene. Nude mice were cultured in SPF environment, with 6 nude mice in each group. Control group: LV-si-NC cells were injected subcutaneously. Knocking group: LV-si-circABCB10 cells were injected subcutaneously. A syringe was used to take 100 *µ*l of 5 × 10^6^/ml cells and inject them subcutaneously into nude mice, and the injection site was pressed with cotton swab. The state of nude mice was observed every week, and the mice were killed by carbon dioxide euthanasia 4 weeks later. Subcutaneous tumors were removed from nude mice and their size and weight were measured. The tumor volume (*V*) was calculated as follows: *V* = (*ab*^2^)/2, where “*a*” represents the long diameter of the tumor and “*b*” represents the short diameter of the tumor mass. Subcutaneous tumors were collected; some of them were frozen at −80°C for qRT-PCR assay, and the other part was stored in formaldehyde for IHC assay. The study was approved by the Ethics Committee of the Fourth Affiliated Hospital of China Medical University.

### 2.11. Immunohistochemical Assay

Standard immunoperoxidase assay was used for staining. The formalin-fixed and paraffin-embedded tissue specimens were cut into sections of 4 *μ*m. It then detaches in xylene and hydrated in alcohol. Incubate with fresh 3% hydrogen peroxide at room temperature for 25 min. Serum-containing PBS was used to block nonspecific antigens in the sections. The diluted antibody (1 : 50) was dropped into sections. Incubate at 4°C overnight, add the secondary antibody, and stain and take photos.

### 2.12. Dual Luciferase Reporter Gene Assay

The cells were washed twice with PBS and digested with trypsin for 2-3 min. Centrifuge at 800 rpm for 5 min; resuspend and mix evenly and spread into 96-well plates (about 10^4^/well). circABCB10-WT, circABCB10-MT, miR-NC, and hsa-miR-1252-5p mimics were transfected, respectively. The transfection reagent was added and incubated for 48 h. The reporter gene lysate was mixed and added into 96-well plate about 100 *μ*L/well. The cells were fully lysed and centrifuged at 10000 g for 5 min. The supernatant was taken for subsequent determination. The amount of luciferase working solution was calculated at 100 *μ*L per sample (the ratio was 1 : 100). The multifunctional enzyme plate analyzer was adjusted to determine the interval of 2 s and the determination time of 10 s. 100 *μ*L of the test sample was added with 100 *μ*L of firefly luciferase reagent, and the fluorescence intensity was measured after mixing the two. Blank control was reporter gene cell lysate. After the above determination, 100 *μ*L of luciferase working solution was added and the fluorescence intensity was measured after mixing. The fluorescence value measured by firefly luciferase was divided by the fluorescence value measured by luciferase (internal reference), and the ratio was obtained to calculate the activation value of reporter genes among different samples.

### 2.13. Fluorescence In Situ Hybridization

Cell slides were placed in 24-well plates, and cells in good growth state were resuspended and mixed evenly and spread into 24-well plates. The degree of cell fusion reached 60–70% before the experiment. The cells were washed with PBS for 5 min and fixed with paraformaldehyde for 10 min. Add 1 mL of permeable solution to each well of the 24-well plate at 4°C for 5 min. The permeable liquid was discarded and washed with PBS for 5 min, 3 times in total. Add 200 *μ*L prehybridization solution into the well at 37°C for 30 min. In the dark chamber, add Fish Probe storage solution into the hybrid solution. The prehybridization solution was discarded, and 100 *μ*L of the hybridization solution containing the probe was added and placed in a dark chamber at 37°C overnight. In the darkroom, DAPI was used for dyeing for 10 min, and PBS was used for washing for 3 times, 5 min each time. In the darkroom, the slide is fixed on the slide with the sealing reagent. Fluorescence microscope was used to detect the results of the sliver.

### 2.14. RNA Pull-Down Experiment

After collection, cells were lysed and treated with ultrasound. The cells were centrifuged at 1000 r/min for 5 min. Transfer the supernatant to the enzyme-free EP tube. A biotin-labeled probe (40 *μ*L, 100 nmol/mL) and streptavidin magnetic beads (40 *μ*L) were added to a centrifuge tube containing a low-salt buffer solution. It was incubated at room temperature for 2 h to form the probe-magnetic bead complex. The probe-magnetic bead complex was separated by a separator. Clean the beads, separate the beads, and remove the liquid. Repeat this step three times. The supernatant of the cell lysate was then incubated with the probe-magnetic bead complex at 4°C overnight. After elution, the magnetic bead binding RNA was extracted with RNeasy mini kit and qRT-PCR was performed.

### 2.15. Western Blot

Gastric cancer cells MKN-45 and MKN28 were seeded into 6-well plates, respectively. Proteins were extracted 48 h after transfection. SDS-PAGE was performed and then transferred to PVDF membrane. BSA was used to seal for 1 h. After sealing, the sealing solution was discarded and the PVDF membrane was cleaned with TBST solution on a decolorizing shaker. The PVDF membrane was placed in the corresponding pre-prepared primary antibody solution. Incubate overnight at 4°C. The PVDF membrane was cleaned with TBST solution for 4 times. After 7 min, the TBST solution was discarded and the corresponding secondary antibody solution was added. Incubate at room temperature for 50 min, and then wash with TBST shaker for 4 times, 7 min each time. ECL luminescent solution was prepared according to the ratio of liquid A:liquid *B* = 1 : 1, and the membrane was placed in the luminescent instrument. Evenly add luminescent solution to the film, adjust the exposure time, and take photos and save them. The absorbance of protein bands was quantitatively analyzed by ImageJ.

### 2.16. Statistical Analysis

SPSS22.0 statistical software was used for data analysis. The results are expressed as mean ± standard deviation. Each experiment was repeated three times. The two-tailed unpaired Student's *t*-test was used to comparisons between two groups. Comparisons among multiple groups were analyzed using one-way ANOVA followed by Tukey's post hoc test. *P* < 0.05 was considered statistically significant.

## 3. Results

### 3.1. circABCB10 Was Highly Expressed in Gastric Cancer Tissues and Cells

**Figure 1:**
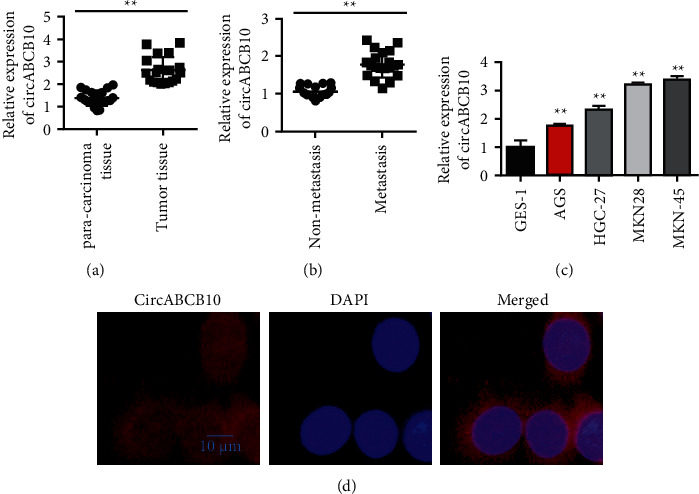
The expression of circABCB10 was upregulated in gastric cancer tissues and correlated with the prognosis of gastric cancer patients. (a) The expression level of circABCB10 in tumor tissues and paracancerous tissues of patients with gastric cancer was detected by qPCR. (b) qRT-PCR results showed that circABCB10 was upregulated in gastric cancer cell lines. (c) circABCB10 is upregulated in patients with gastric cancer metastasis than nonmetastasis patients. (d) Fluorescence in situ hybridization experiment detected the intracellular expression and localization of circABCB10. ^∗∗^*P* < 0.01.

In this study, freshly resected gastric cancer tissues and matched normal gastric mucosa tissues were collected. After RNA extraction, the expression of circABCB10 in each tissue sample was detected by qRT-PCR. Expression differences of circABCB10 in gastric cancer tissues and matched normal gastric mucosa tissues were analyzed. The role and molecular mechanism of circABCB10 in the pathogenesis, invasion, and metastasis of gastric cancer have been explored. The results showed that the expression level of circABCB10 in gastric cancer tissues was significantly higher than that in the matched normal gastric mucosa tissues (Figure 1(a)). Further clinicopathological analysis showed that the expression level of circABCB10 in patients with gastric cancer metastasis was higher than that in patients without metastasis (Figure 1(b)). Subsequently, we detected the expression and localization of circABCB10 in gastric cancer cells. circABCB10 expression was detected by qRT-PCR in a variety of gastric cancer cells (MGC803, MKN-45, MKN28, and MKN-45) and human gastric mucosal epithelial cell GES-1. The experimental results showed that the expression of circABCB10 was higher in gastric cancer cells than in GES-1 cells (Figure 1(c)). FISH assay showed that circABCB10 was mainly localized in the cytoplasm of gastric cancer cells (Figure 1(d)).

### 3.2. Effects of Knockdown of circABCB10 on Proliferation, Invasion, Clone Formation, and Apoptosis of Gastric Cancer Cells

**Figure 2:**
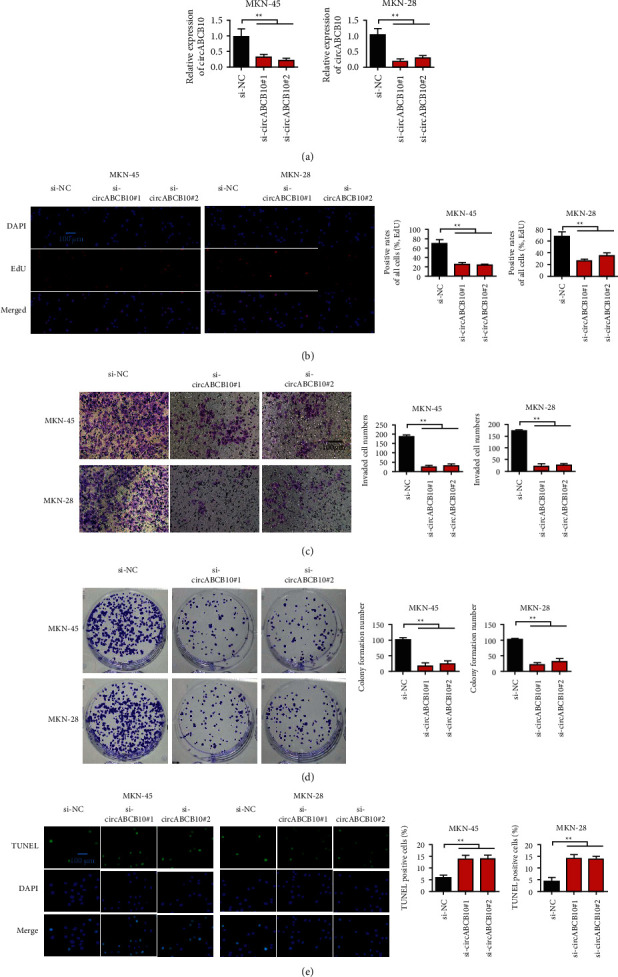
At the cellular level, knockdown of circABCB10 was shown to inhibit malignant behavior in gastric cancer cells. (a) Detection of circABCB10 expression in MKN-45 and MKN28 cells. (b) The effect of circABCB10 on the proliferation of MKN-45 and MKN28 was detected by EdU. (c) Transwell examined the effect of circABCB10 on the invasion ability of MKN-45 and MKN28. (d) The effect of circaBCB10 on the clone formation ability of MKN-45 and MKN28 was detected. (e) TUNEL detected the effect of circABCB10 on apoptosis of MKN-45 and MKN28 cells. ^∗∗^*P* < 0.01.

MKN-45 and MKN28 cells were transfected, respectively. As shown in Figure 2(a), mRNA expression levels of circABCB10 in MKN-45 and MKN28 cells were significantly decreased after si-circABCB10 transfection compared with si-NC group. The above transfected gastric cancer cell lines were used to detect the effect of knockdown of circABCB10 on the proliferation of gastric cancer cells. The proliferation ability of gastric cancer cells was measured by EDU experiment. The results showed that circABCB10 knockdown significantly reduced the proliferation of MKN-45 and MKN28 cells compared with the si-NC group (Figure 2(b)). In this study, Transwell experiment was conducted to investigate the effect of circABCB10 knockdown on the invasion ability of gastric cancer cells. The results showed that knockdown of circABCB10 significantly inhibited the number of cells invaded by MKN-45 and MKN28 cells into the lower Transwell compartment. It was shown that circABCB10 knockdown reduced the invasion ability of MKN-45 and MKN28 cells (Figure 1(c)). In this study, the effect of knockdown of circABCB10 on the clonal formation ability of gastric cancer cells was further detected by clonal formation assay. The results showed that knockdown of circABCB10 significantly reduced the number of clones formed by MKN-45 and MKN28 cells compared with the si-NC group. It was shown that circABCB10 knockdown reduced the ability of MKN-45 and MKN28 cells to form clones (Figure 1(d)). The TUNEL assay was used to detect the effect of circABCB10 knockdown on the apoptosis of gastric cancer cells. The results showed that knockdown of circABCB10 significantly increased apoptosis in MKN-45 and MKN28 cells compared with the si-NC group. It was shown that circABCB10 knockdown increased apoptosis in MKN-45 and MKN28 cells (Figure 1(e)).

### 3.3. Effect of circABCB10 Knockdown on Gastric Cancer Cell Transplantation Tumor

**Figure 3:**
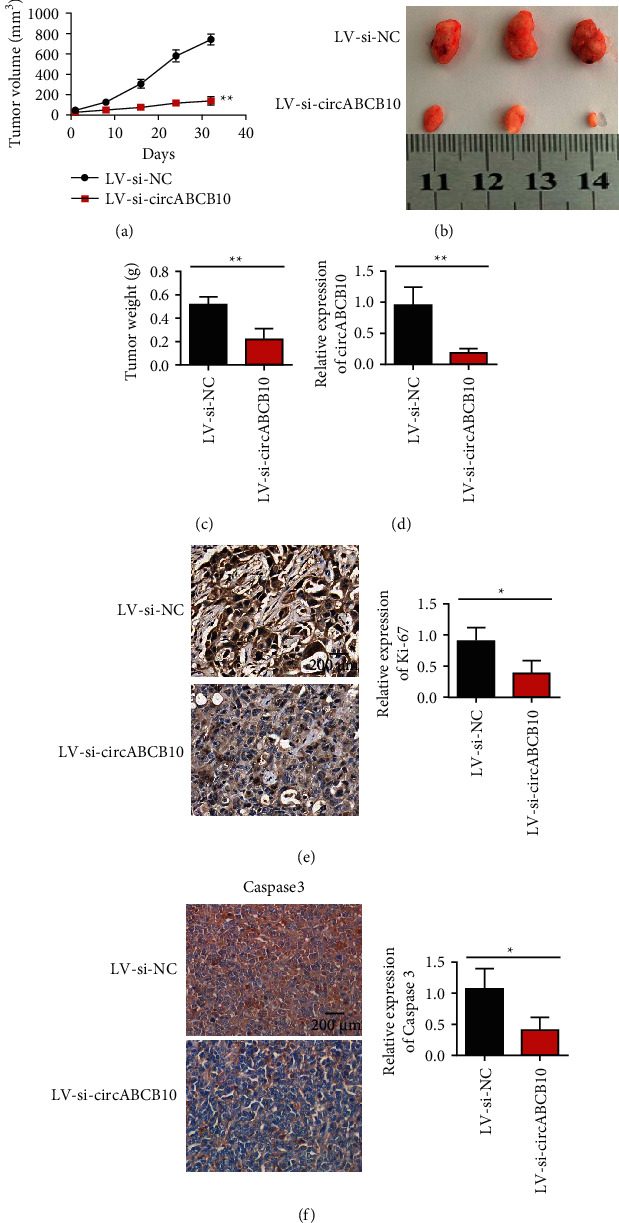
Subcutaneous tumor-bearing experiments in nude mice demonstrated that circaBCB10 knockdown inhibited the proliferation of gastric cancer cells. (a) Tumor proliferation curve in nude mice after different treatments. (b) Tumor weight in nude mice. (c) General picture of the tumor in nude mice. (d) Detection of circABCB10 expression levels in two groups of tumor tissues. (e) Ki-67 immunohistochemical staining. (f) Caspase 3 immunohistochemical staining in control and treated groups. ^∗∗^*P* < 0.05; ^∗∗^*P* < 0.01.

In this study, the effect of circABCB10 knockdown on the proliferation of gastric cancer cells MKN-45 in vivo was investigated by subcutaneously inoculating tumor cells into nude mice. As shown in Figure 3(a), knockdown of circABCB10 significantly inhibited tumor growth in nude mice compared with the empty vector group (Figures 3(a) and 3(b)). Tumor weight test results also showed that knocking down circABCB10 reduced tumor weight (Figure 3(c)). This suggests that knockdown of circABCB10 inhibits tumorigenesis of gastric cancer cells MKN-45 in vivo. Further, we detected the changes in the expression of circABCB10 in tumor tissues after different treatments. The results showed that the expression level of circABCB10 was significantly decreased in the LV-si-circABCB10 group (Figure 3(d)). These results indicated that LV-si-circABCB10 could significantly decrease the expression of circABCB10 in cells. We used immunohistochemistry to detect the expression changes of Ki-67 and Caspase3 in different treated tumor groups. Immunohistochemical results showed that the expression of Ki-67 and Caspase3 in MKN-45 cell line was significantly decreased after circABCB10 silencing (Figures 3(e) and 3(f)).

### 3.4. circABCB10 Directly Binds to miR-1252-5p

**Figure 4:**
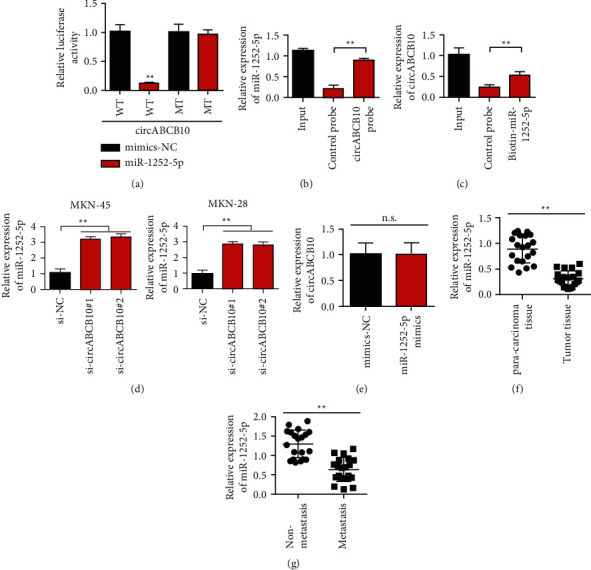
circABCB10 can act as a sponge for miR-1252-5p in gastric cancer cells. (a) Report carrier experiment. (b) RNA pull-down (circABCB10 probe). (c) RNA pull-down (miR-1252-5p probe). (d) Detection of the effect of circABCB10 on miR-1252-5p expression level. (e) Detection of circABCB10 expression level after overexpression of miR-1252-5p. (f) Detection of miR-1252-5p expression levels in adjacent and gastric cancer tissues. (g) Detection of miR-1252-5p expression levels in metastasis and nonmetastasis cancer tissues. ^∗∗^*P* < 0.01.

In this study, we used bioinformatics tools StarBase interactions online platform (http://starbase.sysu.edu.cn/) to predict circABCB10 targeted microRNAs. As shown in Figure 4(a), the results of the dual luciferase reporter gene assay system showed that miR-1252-5p mimic could significantly reduce the luciferase activity of the vector containing the full-length sequence of circABCB10 (circABCB10-WT). miR-1252-5p mimic had no effect on luciferase activity of sequence vector containing circABCB10 mutation site (circABCB10-MT) (*P* > 0.05). Subsequently, we performed RNA pull-down experiment and detected the expression level of miR-1252-5p by qRT-PCR. The results showed that the circABCB10 probe could enrich miR-1252-5p compared with the control probe (Figure 4(b)). Similarly, compared with the control probe, the miR-1252-5p probe was also enriched in circABCB10 (Figure 4(c)). The above experiments proved that circABCB10 directly interacted with miR-1252-5p. Further detection results showed that knockdown of circABCB10 could upregulate the expression of miR-1252-5p in MKN-45 and MKN28 cells (Figure 4(d)). However, the overexpression of miR-1252-5p had no significant effect on the expression of circABCB10 (Figure 4(e)). The expression of miR-1252-5p in gastric cancer and matched normal gastric mucosa was detected by qRT-PCR. The results showed that the expression level of miR-1252-5p in gastric cancer tissues was significantly decreased compared with matched normal gastric mucosa tissues (Figure 4(f), *P* < 0.01). In addition, the expression of miR-1252-5p is downregulated in patients with cancer metastasis (Figure 4(g), *P* < 0.01).

### 3.5. miR-1252-5p Can Directly Bind to MYC

**Figure 5:**
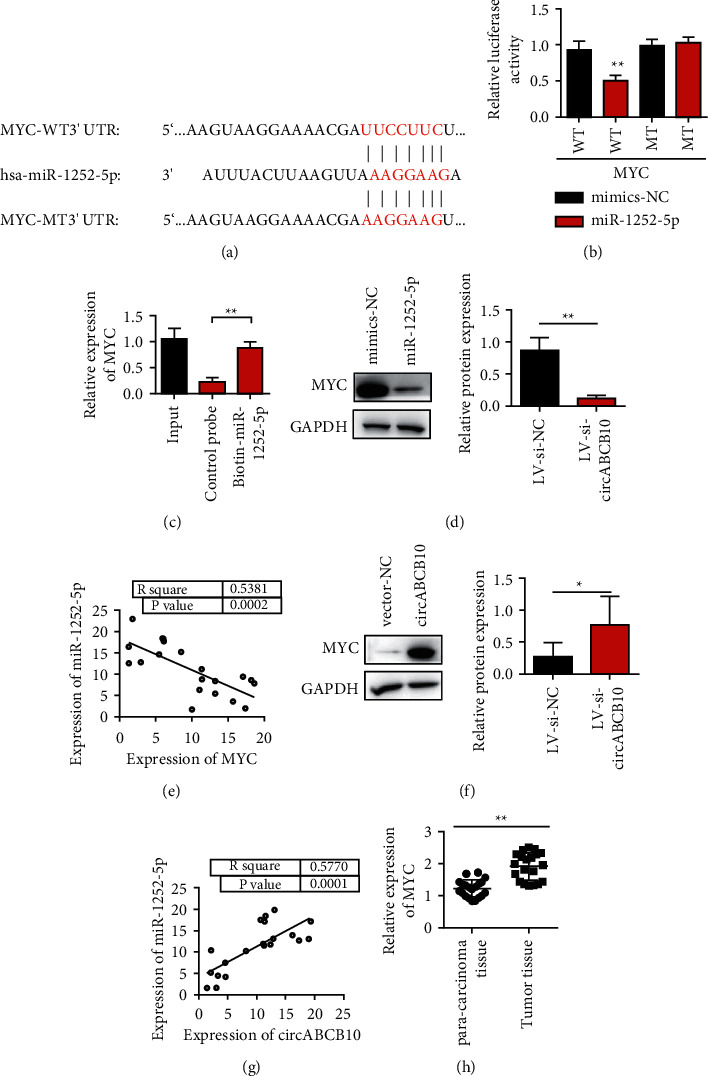
miR-125a-5p targets to bind to MYC. (a) MYC site information targeted by miR-1252-5p. (b) The dual luciferase reporter assay confirmed the binding of miR-1252-5p and MYC. (c) RNA pull-down to detect whether miR-1252-5p binds to MYC. (d) Expression of MYC was inhibited by miR-1252-5p in Western blot detection. (e) Correlation analysis of coexpression of miR-1252-5p and MYC. (f) circABCB10 promoted MYC expression by Western blot detection. (g) Correlation analysis of circABCB10 coexpression with MYC. (h) Expression level of MYC in adjacent and gastric cancer tissues was detected by qRT-PCR. ^∗∗^*P* < 0.05; ^∗∗^*P* < 0.01.

In this study, we use online bioinformatics tools TargetScan website (http://www.targetscan.org/vert_71/) to predict miR-1252-5p targeted MYC.  Figure 5(a) shows the interaction sites between miR-1252-5p and MYC. Further results of dual luciferase reporter gene assay system showed that miR-1252-5p mimic could significantly reduce luciferase activity of vector containing MYC full-length sequence (MYC-WT). miR-1252-5p mimic had no effect on luciferase activity of sequence vector containing MYC mutation site (MYC-MT) (Figure 5(b), *P* > 0.05). The results of the RNA pull-down experiment showed that compared with the control probe, the miR-1252-5p probe could enrich MYC (Figure 5(c)). The above experiments proved that there was a direct interaction between MYC and miR-1252-5p. Western blot results showed that the overexpression of miR-1252-5p could inhibit the expression of MYC (Figure 5(d)). The Pearson correlation test was used to evaluate the correlation between miR-1252-5p and MYC mRNA expression in 20 cases of gastric cancer. The results showed that the expression of miR-1252-5p was negatively correlated with MYC in gastric cancer tissues, *r* =  −0.5381, *P*=0.0002 (Figure 5(e)). Western blot results showed that circABCB10 overexpression upregulated the expression of MYC (Figure 5(f)). Pearson's correlation test was used to evaluate the correlation between the mRNA expression of circaCB10 and MYC in 20 cases of gastric cancer. The results showed that there was a positive correlation between the expression of circABCB10 and MYC in gastric cancer tissues, *r* = 0.577, *P*=0.0001 (Figure 5(g)). The expression of MYC in gastric cancer and matched normal gastric mucosa was detected by qRT-PCR. The results showed that the expression level of MYC in gastric cancer tissues was significantly increased compared with the matched normal gastric mucosa tissues (Figure 5(h), *P* < 0.01).

### 3.6. Overexpression of miR-1252-5p Reversed the Upregulation Effect of circABCB10 on MYC

**Figure 6:**
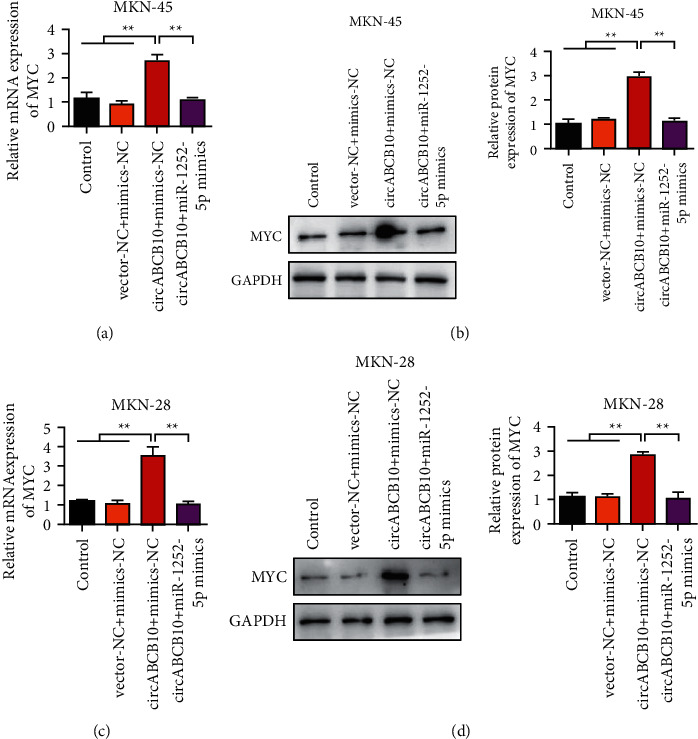
Overexpression of miR-1252-5p reverses the upregulation effect of circABCB10 on MYC. (a) Reverse of the upregulation effect of circABCB10 on MYC by miR-1252-5p in MKN-45 cells was verified by qRT-PCR. (b) Western blot was used to verify the reversal of the upregulation effect of circABCB10 on MYC by miR-1252-5p in MKN-45 cells. (c) Reverse of the upregulation effect of circABCB10 on MYC by miR-1252-5p in MKN28 cells was verified by qRT-PCR. (d) Western blot was used to verify the reversal of the upregulation effect of circABCB10 on MYC by miR-1252-5p in MKN28 cells. ^∗∗^*P* < 0.01.

We verified that miR-1252-5p reversed the upregulation effect of circABCB10 on MYC in MKN-45 cells by qRT-PCR. The qRT-PCR results showed that circABCB10 overexpression could upregulate the expression of MYC. However, the expression of MYC was decreased after the simultaneous transfection of circABCB10 and miR-1252-5p (Figure 6(a)). Western blot results were consistent with qRT-PCR results (Figure 6(b)). Subsequently, we further validated this result in MKN28. Results of MKN28 cells showed that overexpression of miR-1252-5p reversed the upregulation effect of circABCB10 on MYC (Figures 6(c) and 6(d)).

### 3.7. Rescue Experiments Proved That the Overexpression of miR-1252-5p and MYC Knockdown Reversed the Promoting Effect of circABCB10 on Gastric Cancer

**Figure 7:**
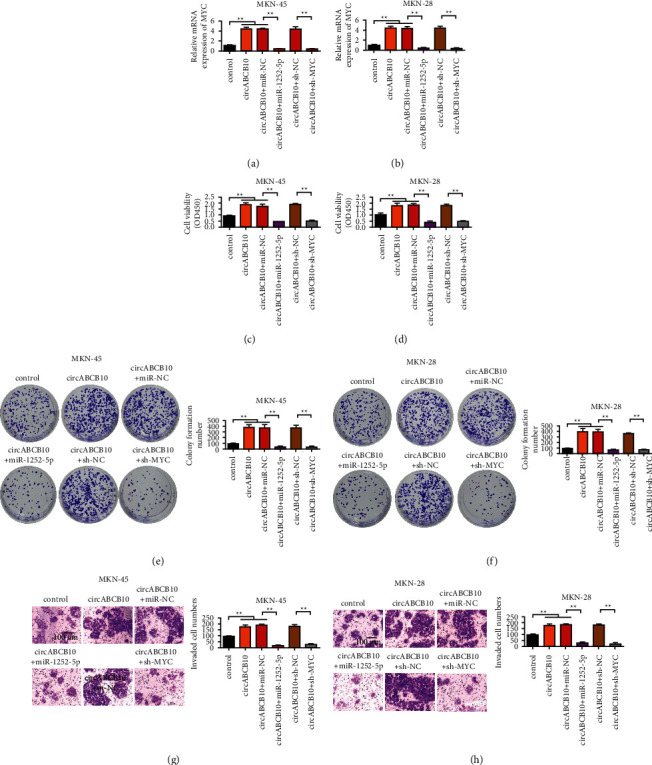
Rescue experiment proved that the overexpression of miR-1252-5p and MYC knockdown reversed the promoting effect of circABCB10 on gastric cancer. (a) Detection of MYC expression level in MKN-45 cells. (b) Detection of MYC expression level in MKN28 cells. (c) CCK-8 assay of MKN-45 cell proliferation. (d) CCK-8 assay of MKN28 cell proliferation. (e) Clonal formation experiment of MKN-45 cells after different treatment. (f) Clonal formation experiment of MKN28 cells after different treatments. (g) Invasion test of MKN-45 cells after different treatments. (h) Invasion test of MKN28 cells after different treatments. ^∗∗^*P* < 0.01.

Transfected human gastric cancer cells MKN-45 and MKN28 were constructed by transfection technique. The transfection efficiency of MYC was shown in Figure S1. The results showed that shRNA MYC decreased the expression of MYC in MKN-45 and MKN28 cells. After 48 hours, qRT-PCR was used to verify the expression of MYC in gastric cancer cell lines transfected with circABCB10, circABCB10 +miR-1252-5p, and circABCB10 + sh-MYC, respectively. The results showed that MYC expression was upregulated after circABCB10 transfection, and the difference was statistically significant. However, the expression of MYC was decreased after simultaneous transfection of circABCB10 and miR-1252-5p. Similarly, compared with circABCB10, MYC expression was decreased after simultaneous transfection of circABCB10 + sh-MYC (Figures 7(a) and 7(b)). After 72 h of culture, the proliferation ability of MKN-45 and MKN28 cell lines was detected by CCK-8 assay. The absorbance value of circABCB10 transfected group was higher than that of control group, and the difference was statistically significant. The absorbance values of MKN-45 and MKN28 cells were decreased after simultaneously transfecting circABCB10 + miR-1252-5p and circABCB10 + sh-MYC compared with the circABCB10 group (Figures 7(c) and 7(d)). Transwell test was used to compare the invasiveness of cells in each group. The number of transfected cells in circABCB10 group was higher than that in control group, and the difference was statistically significant. Compared with circABCB10, circABCB10 + miR-1252-5p and circABCB10 + sh-MYC simultaneously transfected MKN-45 and MKN28 cells reduced the number of transmembrane cells (Figures 7(e) and 7(f)). The results of clone formation ability test were consistent with Transwell results. The number of clone cells in circABCB10 transfected group was higher than that in control group, and the difference was statistically significant. Compared with circaBCB10, circaBCB10 + miR-1252-5p and circaBCB10 + sh-MYC simultaneously transfected MKN-45 and MKN28 cells showed a reduced number of cloned cells (Figures 7(g) and 7(h)). It is suggested that the simultaneous overexpression of miR-1252-5p or downregulation of MYC in MKN-45 and MKN28 cell lines can reverse the cancer-promoting effect of circABCB10.

## 4. Discussion

Gastric cancer is one of the most common gastrointestinal tumors in the world [14, 15]. Although the incidence and mortality of gastric cancer have decreased in recent years, the 5-year survival rate of early gastric cancer after radical surgery and related adjuvant therapy can reach more than 90%. However, because the symptoms of early gastric cancer are not obvious and screening for gastric cancer is not widespread, once diagnosed, most of them are in the middle and late stages and have lost the opportunity of surgery [16, 17]. Even in patients after surgery, recurrence and mortality are still high [18]. In this study, we focused on a novel gene regulator, circRNA. circRNAs are special noncoding RNAs that are widely found in organisms [19]. It is not only closely related to a variety of diseases, but also has potential value in cancer diagnosis and biological therapy [20]. Studies have shown that a variety of malregulated circRNAs have been found in gastric cancer tissues, cells, and even plasma of patients with gastric cancer [21–23]. At present, circRNA has been studied in a variety of malignant tumors, and circRNA is also closely related to bladder cancer [24], esophageal cancer [25], cervical cancer [26], oral cancer [27], and other tumors. In gastric cancer, Li et al. reported that circ_002059 was abnormally low expressed in gastric cancer tissues and suggested that it might be a potential tumor marker for early diagnosis of gastric cancer [20]. Lu et al.'s study showed that circ_0000467 was abnormally high expressed in gastric cancer tissues and serum of gastric cancer patients, and the expression level of circ_0000467 was significantly correlated with TNM staging of gastric cancer patients. The expression level of circ_0000467 in patients' serum after surgery was significantly lower than that before surgery, suggesting that circ_0000467 could be used as a new noninvasive biomarker for the diagnosis and prognosis of gastric cancer [28]. At present, the research related to circRNA and gastric cancer is still in the initial stage and mainly focuses on the detection of circRNA expression in tumor samples, and only a few studies have explored the function and mechanism of action of circRNA in tumors.

In this study, circABCB10 was used as the research object. First, we performed qRT-PCR assay to analyze the expression level of circABCB10 in gastric cancer. The results showed that circABCB10 was significantly overexpressed in gastric cancer tissues and cells. After knockdown of circABCB10 by RNA interference technology, the experiments of proliferation, invasion, and clone formation were carried out. The results showed that the proliferation, invasion, and clone formation ability of gastric cancer cells MKN-45 and MKN28 were decreased when circABCB10 was low expressed. This suggested that circABCB10 could affect the biological function of gastric cancer.

The role of miRNA in cancer has been studied extensively [29]. Various abnormally expressed miRNAs have been found to be associated with the progression and prognosis of gastric cancer. It has been reported that miR-1252-5p enhances the sensitivity of bortezomib in multiple myeloma cells by targeting heparin [30]. In addition, miR-204 has also been found to inhibit the proliferation of gastric cancer cells by targeting CKS1B, CXCL1, and GPRC5a [31]. Most circRNA molecules contain miRNA response elements and can bind to miRNAs. For example, Zeng et al. [32] found that circHIPK3 can adsorb miR-7 to promote the growth and metastasis of colorectal cancer. Xie et al. found that circrNABCRC-3 acted as a tumor suppressor by inhibiting the proliferation of bladder cancer cells through the miR-182-5p axis [33]. Based on the characteristics of circRNA and miRNA, we predicted that miR-1252-5p could interact with circABCB10 and conducted the dual luciferase assay by constructing dual luciferase reporter plasmid containing wild-type and mutant circABCB10 to interact with overexpressed miR-1252-5p. The results showed that the overexpression of miR-1252-5p inhibited luciferase activity of wild-type circABCB10. However, it did not inhibit luciferase activity of the mutant circABCB10. These results suggested that circABCB10 acted as a sponge adsorber for miR-1252-5p in gastric cancer cells.

C-MYC is a transcription factor encoded by the MYC protooncogene [34]. Its protein product is a DNA-binding phosphoprotein, which is an important transcriptional activator in the regulation of normal cell growth and differentiation, and its expression regulation plays a key role in the balance of cell proliferation and differentiation. It is overexpressed in various malignant tumors such as breast cancer, liver cancer, and pancreatic cancer [35, 36], which is consistent with the findings in this study that the expression of c-MYC is significantly upregulated in both gastric cancer tissue specimens and cell lines and is consistent with the results related to the degree of invasion of gastric cancer. We further observed the expression of c-MYC in different groups. Results showed that knockdown of circABCB10 reduced MYC expression in MKN-45 and MKN28. miR-1252-5p reversed this effect. Some studies have reported that other miRNAs can regulate MYC and further affect the occurrence and development of gastric cancer. The expression of miR-145 was significantly downregulated in gastric cancer tissues, and the transfection of miR-145 could inhibit the activity of gastric cancer cell lines. Further experiments confirmed that miR-145 can regulate c-MYC expression in gastric cancer cell lines [37].

In this study, we found that circABCB10 was highly expressed in gastric cancer and could play a role in promoting cancer. In addition, circABCB10 acted as a sponge for miR-1252-5p and affected the proliferation, invasion, and apoptosis of gastric cancer cells. There are also shortcomings in this study. Due to the short study time, we did not study the association between the expression level of circABCB10 and patient survival. Through further studies, we will have a deeper understanding of circABCB10, which is expected to become a molecular marker for the diagnosis of gastric cancer, and silencing circABCB10 may become a new method of targeted therapy for gastric cancer.

## 5. Conclusion

In this study, we found that circABCB10 was highly expressed in gastric cancer tissues and gastric cancer cells and was associated with metastasis of gastric cancer. We further confirmed that downregulation of circABCB10 expression could significantly inhibit the proliferation and invasion of gastric cancer cells. Bioinformatics analysis and dual luciferase reporter gene detection confirmed that circABCB10 and miR-1252-5p had a highly effective binding site. Further, we found that MYC was the target gene of miR-1252-5p. At the same time, the co-downregulation of circABCB10 and miR-1252-5p could reverse the decreased proliferation and invasion ability of MKN-45 and MKN28 cells induced by circABCB10 knockdown. These results suggest that circABCB10 is involved in the regulation of the biological functions of gastric cancer cells and is closely related to tumor proliferation and invasion. The results of this study suggest that targeting circABCB10 may become a new strategy for the treatment of gastric cancer.

## Data Availability

The datasets used and/or analyzed during the current study are available from the corresponding author upon reasonable request.

## Supplementary Materials

Supplementary Materials
*Figure S1*. The transfection efficiency of shRNA MYC in MKN-45 and MKN28 cells.
