# Supplementary figures and images for: circABCB10 Promotes Malignant Progression of Gastric Cancer Cells by Preventing the Degradation of MYC
Source: J Oncol. 2021 Dec 14;2021:4625033. doi: 10.1155/2021/4625033 (PMC8692003; doi:10.1155/2021/4625033)

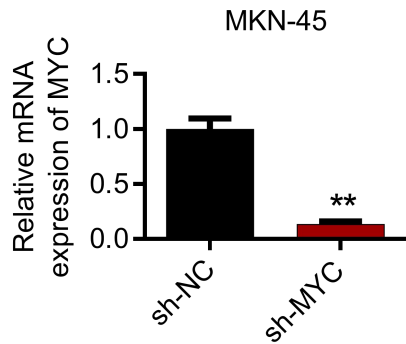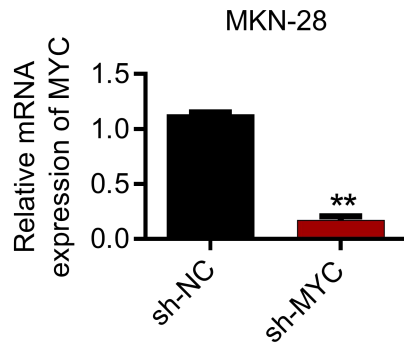

Supplement: Supplementary Materials — Figure S1. The transfection efficiency of shRNA MYC in MKN-45 and MKN28 cells. [file 4625033.f1.pdf]
